# Role of Cytokines During Epileptogenesis and in the Transition from the Interictal to the Ictal State in the Epileptic Mutant EL Mouse

**Published:** 2008-08-27

**Authors:** Yoshiya L. Murashima, Jiro Suzuki, Mitsunobu Yoshii

**Affiliations:** Division of Psychobiology, Tokyo Institute of Psychiatry, Tokyo, Japan

**Keywords:** cytokines, epileptic mutant, EL mouse, epileptogenesis, transition, interictal state, ictal state

## Abstract

**Purpose:**

Epileptic mutant EL mice show secondary generalized seizures. Seizure discharges initiate in the parietal cortex and generalize through the hippocampus. We have previously demonstrated an increase in the activity of inducible nitric oxide synthetase (iNOS) as well as a decrease in the activity of superoxide dismutase (SOD) in the hippocampus of EL mice, suggesting that cell toxic free radicals are increased in the brain of EL mice. In parallel with this, neurotrophic factors were significantly increased in the hippocampus of EL mice in earlier developmental stages before exhibiting frequent seizures. These findings were no longer present after frequent seizures, suggesting that these events may trigger ictogenesis. On the other hand, it is reported that limbic seizures rapidly induce cytokines and related inflammatory mediators. It remains to be seen, however, whether cytokines contribute to the transition from interictal to ictal state. The present study was designed to address this issue using EL mice.

**Methods:**

EL mice at the age from 4 to 23 weeks and their control animal, DDY mice at the age of 10 and 20 weeks were used. Seizures were induced in EL mice once every week since 5 weeks. Cytokines, such as interleukin-1 alpha (IL-1a), interleukin 1-beta (IL-1b), IL-6, IL-1 receptor (IL-1r), IL-1 receptor antagonist (IL-ra) and tumor necrosis factor alpha (TNF-a) were examined by Western blotting in the ‘focus complex’ of brain (namely, in the parietal cortex and hippocampus) of EL mice in the interictal period at different developmental stages. In 15 week old EL mice, which show seizures once a week, these cytokines were similarly determined 5 min, 2 hr, 4 hr, 11 hr, 24 hr, 3 days and 6 days after the last seizure induced.

**Results:**

A significant increase in the level of cytokines was observed in the brain of EL mice at any stages during development, compared with the level of cytokines in the brain of control DDY. Cytokines were increased predominantly before experiencing frequent seizures. In EL mice at the age of 15 weeks, the level of cytokines in the hippocampus was highest 6 days after seizures. In the parietal cortex, cytokines were most highly expressed 2 hr after seizures. The results indicate that cytokines were kept up-regulated until next seizures in the hippocampus, whereas they were transiently up-regulated immediately after seizures in the parietal cortex.

**Conclusion:**

It is concluded that in the brain of EL mice, pro-inflammatory cytokines are increased progressively and periodically in association with the development and the seizure activity, respectively. A periodic increase of cytokines prior to the next seizure episode may play a role in triggering the ictal activity. Namely, alteration of region-specific cytokines may induce ictal activities from the interictal state. It is conceivable that inflammatory cytokines may work together with neuronal factors during epileptogenesis and in the transition from interictal to ictal state.

## Introduction

Epileptic mutant EL mice show secondary generalized seizures (1, 2). Seizure discharges initiate in the parietal cortex and generalize through the hippocampus (3). We have previously demonstrated an increase in the activity of inducible nitric oxide synthetase (iNOS) (4) as well as a decrease in the activity of Cu, Zn-superoxide dismutase (Cu, Zn-SOD) (5) in the hippocampus of EL mice, suggesting that cell toxic free radicals are increased in the brain of EL mice.

In parallel with this, neurotrophic factors, such as brain-derived neurotrophic factor (BDNF) and neurotrophin-3 (NT-3), were significantly increased in the hippocampus of EL mice in earlier developmental stages before exhibiting frequent seizures (6). These findings were no longer present after frequent seizures, suggesting that these events may trigger ictogenesis.

On the other hand, it is reported that limbic seizures rapidly induce cytokines and related inflammatory mediators (7). It remains to be seen, however, whether cytokines contribute to the transition from interictal to ictal state. The present study was designed to address this issue using EL mice.

## Material and Methods

EL mice at the age from 4 to 23 weeks and their control animal, DDY mice at the age of 10 and 20 weeks were used. Seizures were induced in EL mice once every week (once every 7 days) since 5 weeks. Cytokines, such as interleukin (IL)-1 alpha (IL-1a), IL-1 beta (IL-1b), IL-6, IL-1 receptor (IL-1r), IL-1 receptor antagonist (IL-1ra) and tumor necrosis factor-alpha (TNF-a) were examined by Western blotting in the ‘focus complex’ of brain (namely, in the parietal cortices and the hippocampus) of EL mice in the interictal period at different developmental stages. In 15 week old EL mice, which show seizures once a week regularly, these cytokines were similarly determined 5 min, 2 hr, 4 hr, 11 hr, 24 hr, 3 days and 6 days after the last seizure induced.

### EL mice

The EL mouse is an inbred mutant strain, which has been used as an animal model of secondarily generalized seizures. (1, 2). *And the mechanism of induction of seizures is the stimulation of vestibular system by tossing-up stimulation or picking up and tail rotating stimulation (1).* The mode of inheritance is autosomal dominant and the penetration rate is 100%. Even before exhibiting seizures, the first seizure discharges will be recorded from the brains of EL mice at the age of over 10 weeks. The mutant animals show frequent seizures after 20 week old.

Several lines of evidence indicate that seizure discharges are initiated in the parietal cortex, and then spread through the hippocampus (3). These findings have been substantiated by the histo-chemical and biochemical analyses of glucose utilization (8) and study of inhibitory neurotransmission mediated by γ-aminobutyric acid (GABA) (9). The developmental formation of the focus complex (epileptogenic zone), which mainly consists of the parietal cortex and the hippocampus, has been hypothesized to be key to ictogenesis and epileptogenesis in EL mice (3).

### Preparation of tissues for immunoblotting analysis

For immunoblotting analysis of the cytokine family (IL-1a, IL-1b, TNF-a, IL-6), IL-1r and IL-1ra, EL mice at 4, 6, 8, 10, 12, 15, 19, 23 weeks of age were used (6 animals for each age). Six DDY mice (mother strain of EL) for each at 10, 20 weeks were used as controls. After decapitation, brains were removed, then the hippocampus and the parietal cortex were dissected out. Approximately 10% of brain homogenates were prepared with 20 mM Tris-HCl buffer (pH 8.0). Just before use, homogenates with dye for 15% SDS-PAGE gels were boiled for 3 min, then centrifuged for 5 min at 3000× rpm.

### Primary antibodies and their specificities

Anti-mouse IL-1a and -1b were purchased from Sigma (Saint Louis, USA). The anti-mouse IL-1a antibody is derived in goat using recombinant mouse IL-1a, expressed in *E. coli*, as immunogen. The anti-mouse IL-1b antibody is derived in goat using recombinant mouse IL-1b, expressed in *E. coli*, as immunogen. Anti-mouse TNF-a was also purchased from Sigma. The antibody is developed in goat using recombinant mouse TNF-a (rmTNF-a) expressed in *E. coli*, as immunogen. Rabbit anti-IL-1r polyclonal antibody was purchased from Abcam (Cambridge, UK). The antibody is derived in rabbit using recombinant mouse IL-1r, expressed in *E. coli*, as immunogen. Rat anti mouse CD121a cell surface antigen, also known as the IL-1r type 1 was purchased from Accurate Chemical and Scientific Cooperation (NY, USA). The antibody blocks the binding of IL-1a, IL-1b and IL-1ra to the receptor. Anti-mouse IL-1r antagonist was purchased from R and D systems (MN, USA). The antibody is produced in goats immunized with purified, *E. coli*-derived, recombinant mouse IL-1ra (rmIL-1ra). IL-1ra specific IgG was purified by mouse IL-1ra affinity chromatography. Rabbit polyclonal anti mouse IL-6 was purchased from Abcam (Cambridge, UK). The antibody is raised against aminoacids 30–212 of IL-6 of human origin and react with mouse IL-6.

### Immunoblotting procedures and data analysis

Immunoblotting studies were carried out according to procedures by Arima and Uéda (10), which were the partially modified. For the immunoblot analysis, 10% mouse brain homogenates (total protein: 30.0–31.0 μg) were separated by 15% SDS-PAGE and transferred to a PVDF membrane (Millipore, Bedford, MA, USA), blocked and then incubated with either anti-IL-1a (1:1,000), or IL-1b (1:1,000), or IL-1r (1:500), or IL-1ra (1:500), or TNF-a(1:1,000), IL-6 (1:250) antibody. After washing, the membrane was incubated with rabbit anti-goat, goat anti-rabbit or rabbit anti-rat conjugated with horseradish peroxidase (1:10,000) purchased from Sigma, which was followed by the Western blot-chemiluminescence reagent (DuPont NEN, Boston, USA). The luminescence of the membrane was detected using Hyperfilm-ECL (Amersham, Little Chalfont, UK). The data were semi-quantitatively analyzed by using the NIH Image with macros software.

## Results

In the parietal cortex and the hippocampus of EL mice, a significant increase in the level of cytokines was observed when compared to their control animals, DDY mice, at any stages during development. Cytokines were increased predominantly before experiencing frequent seizures. In the hippocampus of EL mice at the age of 15 weeks when they show seizures once a week, cytokine levels were highest 6 days after the last seizure. In contrast, peak levels of cytokines were observed 2 hr after seizure in the parietal cortex. Cytokines were up-regulated just before seizures in the hippocampus, or just after seizures in the parietal cortex.

**Figure 1:**
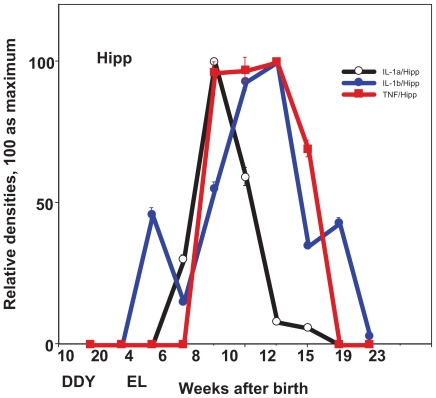
Expression of cytokines during development in the hippocampus (Hipp), seizure generalization and propagation site. Relative expression of IL-1a, IL-1b, TNF-a in control DDY and EL mice during development. Mean ± SEM.

In the seizure initiation site—the parietal cortex (Pcx), the expression of IL-1a gradually increased and reached a peak level at the age of 10 weeks, the time when the first seizure begins and falls. In the hippocampus (Hipp), the expression of IL-1a showed a sharp peak at 8 weeks, or just before the first seizure (Fig. 1, 3). The expression of IL-1b was peak at the age of 10 weeks in Pcx or at the age of 10–12 weeks in Hipp (Fig. 1, 3). The expression of TNF-a shows a relatively high level from 8 weeks to 15 weeks of age in either Pcx or Hipp, although it was up-regulated in Pcx and down-regulated in Hipp (Fig. 1, 3). The expression of monomer IL-6 shows maximum at the age of 10 weeks, however the dimer shows sharp peak at the age of 10 weeks in the Pcx and at the age of 12 weeks in the Hipp (Fig. 5).

**Figure 2:**
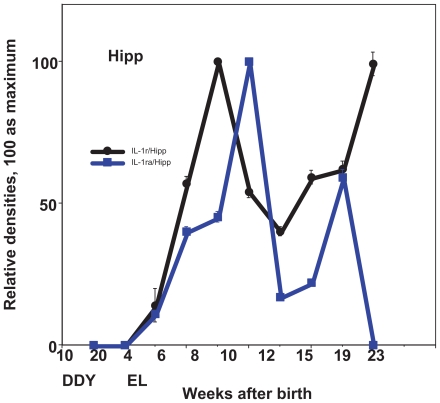
Expression of cytokines during development in the hippocampus (Hipp), seizure generalization and propagation site. Relative expression of IL-1R, IL-1Ra in control DDY and EL mice during development. Mean ± SEM.

**Figure 3:**
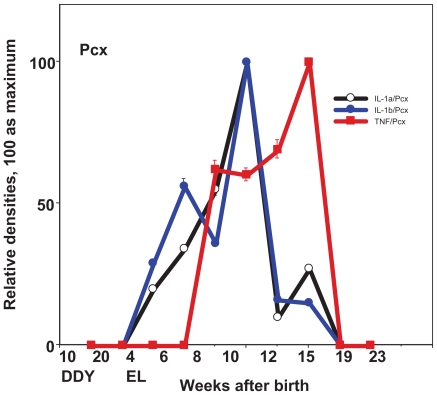
Expression of cytokines during development in the parietal cortex (Pcx), seizure initiation site. Relative expression of IL-1a, IL-1b, TNF-a in control DDY and EL mice during development. Mean ± SEM.

**Figure 4:**
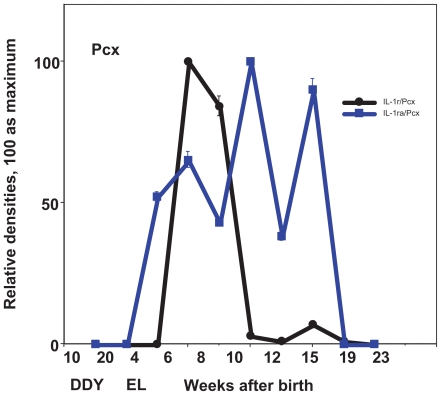
Expression of cytokines during development in the parietal cortex (Pcx), seizure initiation site. Relative expression of IL-1R, IL-1Ra in control DDY and EL mice during development. Mean ± SEM.

In the case of IL-1r, the expression in Pcx showed a sharp peak at the age of 6–8 weeks, whereas the expression in Hipp showed a maximum peak at the age of 8 weeks and kept relatively high levels during frequent seizures (Fig. 2, 4). IL-1ra showed a relatively high expression in either Pcx or Hipp from the early stages before exhibiting the first seizure to the later stages of frequent seizures with a peak at the age of 10 weeks (Fig. 2, 4).

**Figure 5:**
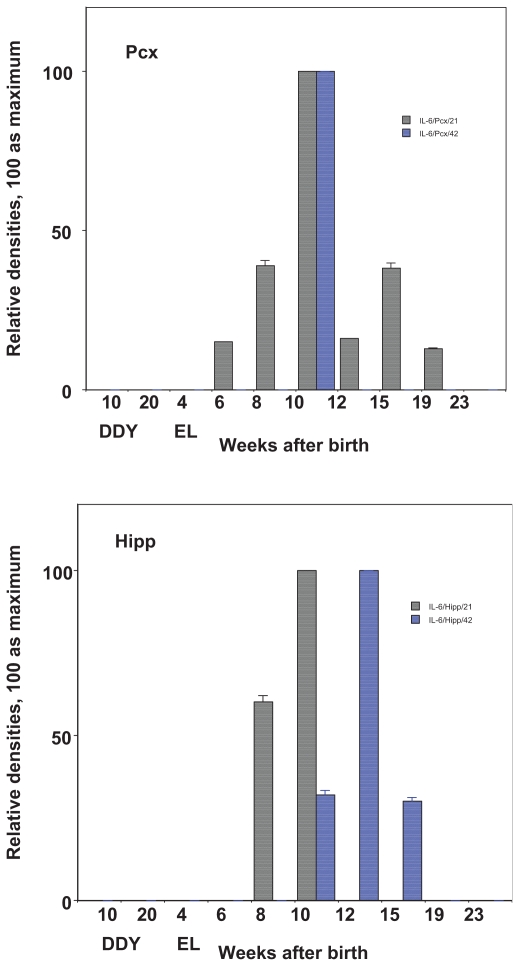
Expression of IL-6 during development in the parietal cortex (Pcx), seizure initiation site and in the hippocampus (Hipp), seizure generalization and propagation site. Relative expression of IL-6 (21KDa monomer and 42KDa dimmer) in control DDY and EL mice during development. Mean ± SEM.

**Figure 6:**
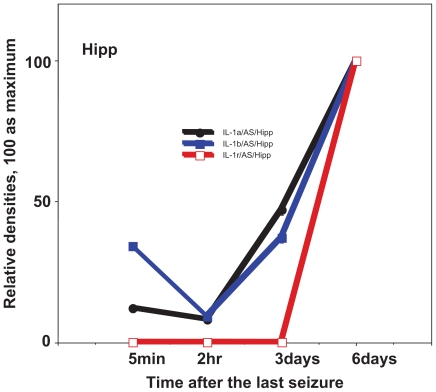
Expression of cytokines after the last seizure in the hippocampus (Hipp) using 15 week old EL mice which show seizure once a week by changing the cage. Relative expression of IL-1a, IL-1b and IL-1R. Mean ± SEM. The physiological meaning of the time course is that in 5 min, EEG has recovered as before seizures; in 2 hrs, absolute refractory period is over; in 3 days, developmental interictal samples were normally obtained; in 6 days, just before the next seizure.

In the seizure propagation site—Hipp, cytokines were expressed at relatively low levels immediately after seizures, whereas they were expressed most strongly just before seizures (Fig. 5). Contrary to Hipp, the seizure initiation site—Pcx showed a maximum expression of cytokines immediately after seizures, then the expression gradually decreased (Fig. 6).

## Discussion

In the present study, a significant increase in the level of cytokines was observed in the brain of EL mice at any stages during development. Cytokines were increased predominantly before experiencing frequent seizures. In EL mice at the age of 15 weeks, the level of cytokines in the hippocampus was highest 6 days after seizures (in which seizures were induced every 7 days). In the parietal cortex, cytokines were most highly expressed 2 hr after seizures. The results indicate that cytokines were kept upregulated until next seizures in the hippocampus, whereas they were transiently upregulated immediately after seizures in the parietal cortex.

In human temporal lobe epilepsy, a variety of cytokines such as IL-1b, IL-6 and IL-ra are upregulated in the cerebrospinal fluid (CSF) in human temporal lobe epilepsy (11). IL-1b shows proconvulsant effects by the intrahippocampal injection using kainate model (7, 12), although the role of IL-1b to epileptogenesis remains unclear. IL-1b and IL-ra during seizures may play a role in altering neuronal network excitability and the associated events (12, 13, 14). In addition, IL-1ra, IL-6, TNF-a show neuroprotective and anticonvulsant actions at low concentrations through blocking IL-1r mediated activity which blocked the neuro-toxic effects of IL-1b (12, 14).

In the present study, IL-1a, IL-1b, IL-6, TNF-a, IL-1R and IL-1Ra in the brain of EL mice were all up-regulated during the epileptogenesis, especially in the early stage before showing frequent seizures. These results suggest that the up-regulation of cytokines in EL mice is associated with triggering of the ictogenesis, rather than as a consequence of frequent seizures.

In EEG recordings of EL, from the pr-eictal state to the ictal state, the initial spike was observed in the parietal cortex (Pcx). Then the spike was propagated to the hippocampus (Hipp) synchronously. And the spikes in the Hipp were generalized and show high amplitudes independent from the initial rhythm of Pcx (1). These findings in EEG suggest that the hyperexcitability initiated in the Pcx is not sufficient enough to show tonic clonic seizures. Once the Hipp is activated independently through the cortico-hippocampal projection, EL mice show clinical manifestation.

In EL mice, an animal model of secondarily generalized seizures, cytokines were induced in both seizure initiation site (parietal cortex) and seizure propagation and generalization site (hippocampus). Cytokines are also induced in fore-brain regions where seizures originate and spread in kainate and bicuculline models (12, 14). Their syntheses in neurons are high when seizure activity is advanced in these models. In EL mice, the peak expression of cytokines was not after frequent seizures. Cytokines may be induced after seizures in chemical models of seizures. In our case using an epileptic mutant, the process of epileptogenesis is presumably established during development. For these reasons, the expression of cytokines may reach a peak before showing frequent seizures.

In the hippocampus of EL mice at the age of 15 weeks when EL mice show seizures once a week (9), the level of cytokines was highest 6 days after the last seizure. In contrast, the peak level of cytokines was observed 2 hr after the last seizure in the parietal cortex. These findings suggest that cytokines were up-regulated just before seizures in the hippocampus, but just after seizures in the parietal cortex.

The role of cytokines in the seizure initiation site and seizure propagation/generalization site may be different.

Roles of cytokines in relation with developmental changes mean the contribution to the establishment of epileptogenesis, however, the roles of cytokines in relation with epileptogenic changes mean the contribution to the transition from the pre-ictal state to the ictal state. The roles for the latter phenomena are the trigger of repeated transient membrane hyperexcitability. But the roles for the former phenomena are to maintain the hyperexcitability of epileptic neural network.

Several reports are available for the up-regulation of cytokines after the seizures. However, it is logically impossible how to determine cytokine expression levels just before the last seizure using a chemical model of epilepsy. In the epileptic mutant EL mice, twelve babies were borne in the first delivery (9). Six were used for the observation of epileptic behavior and the other six were used for the brain sampling. At the age of 15 weeks, EL mice showed seizures almost once a week. After the latter six were sacrificed, the former six showed seizures on the next day. Sometimes on handling quite a few of the latter group showed seizures. These EL mice were excluded from the sample group. EL mice appear to be a useful model of epilepsy to investigate the transition from the pre-ictal or interictal to the ictal state during development.

As for the meanings of up-regulation of TNF-a, We have observed DNA fragmentation and apoptosis related Bcl-2 family (Bcl-2, Bax, BclXL) expression in the hippocampus of EL at an early stage during development before exhibiting seizures (15). One possibility of up-regulated TNF-a may accellate the apoptotic process with the increased cell toxic free radical environments in the hippocampus (15) and then lead to ictogenesis and/or epileptogenesis.

It is concluded that in the brain of EL mice, pro-inflammatory cytokines are increased progressively and periodically in association with the development and the seizure activity, respectively. A periodic increase of cytokines prior to the next seizure episode may play a role in triggering the ictal activity. Namely, alteration in region-specific cytokines may induce ictal activities from the interictal state. It is conceivable that inflammatory cytokines may work together with neuronal factors during epileptogenesis and in the transition from interictal to ictal state.

## Figures and Tables

**Figure 7:**
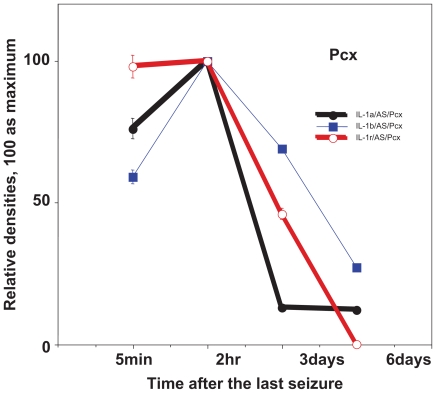
Expression of cytokines after the last seizure in the parietal cortex (Pcx) using 15 week old EL mice, which show seizures once a week by changing the cage. Relative expression of IL-1a, IL-1b and IL-1R. Mean ± SEM.
